# Lea’s Medical Epitome Series—Diseases of the Skin

**Published:** 1903-01

**Authors:** 


### Books and Magazines.

**All books intended for review in the Texas Medical Journal must be sent to Associate Editor Dr. W. B. Russ, San Antonio, Texas.**

Lea's Medical Epitome Series—Diseases of the Skin.—A manual for students and practitioners. By Alfred Schalek, M. D., Instructor of Dermatology, Genito-Urinary and Veneral Diseases, Rush Medical College, Chicago, Illinois. Series edited by C. V. Pederson, A. M., M. D., New York City, N. Y. Illustrated with 24 engravings. Lea Bros. & Co., Philadelphia and New York. 1902. Price, $1.00, net.

This little volume answers all the demands made upon it, and well fills the place for which it is intended. It embodies all the essential features of the various skin diseases, dealing in a short but accurate and comprehensive style with symptomatology, etiology, pathology, diagnosis, treatment and prognosis. L.

